# The rising problem of antimicrobial resistance in the intensive care unit

**DOI:** 10.1186/2110-5820-1-47

**Published:** 2011-11-23

**Authors:** Nele Brusselaers, Dirk Vogelaers, Stijn Blot

**Affiliations:** 1 Department of General Internal Medicine, Infectious Diseases, and Psychosomatic Medicine, Ghent University Hospital, Ghent, Belgium; 2 Faculty of Medicine and Health Sciences, Ghent University, Ghent, Belgium; 3 University College, Ghent, Belgium

## Abstract

Mainly due to its extremely vulnerable population of critically ill patients, and the high use of (invasive) procedures, the intensive care unit (ICU) is the epicenter of infections. These infections are associated with an important rise in morbidity, mortality, and healthcare costs. The additional problem of multidrug-resistant pathogens boosts the adverse impact of infections in ICUs. Several factors influence the rapid spread of multidrug-resistant pathogens in the ICU, e.g., new mutations, selection of resistant strains, and suboptimal infection control. Among gram-positive organisms, the most important resistant microorganisms in the ICU are currently methicillin-resistant *Staphylococcus aureus *and vancomycin-resistant enterococci. In gram-negative bacteria, the resistance is mainly due to the rapid increase of extended-spectrum Beta-lactamases (ESBLs) in *Klebsiella pneumonia*, *Escherichia coli*, and *Proteus *species and high level third-generation cephalosporin Beta-lactamase resistance among *Enterobacter spp*. and *Citrobacter spp*., and multidrug resistance in *Pseudomonas aeruginosa *and *Acinetobacter *species. To conclude, additional efforts are needed in the future to slow down the emergence of antimicrobial resistance. Constant evaluation of current practice on basis of trends in MDR and antibiotic consumption patterns is essential to make progress in this problematic matter.

## Introduction - the burden of multidrug resistance

The intensive care unit (ICU) often is called the epicenter of infections, due to its extremely vulnerable population (reduced host defences deregulating the immune responses) and increased risk of becoming infected through multiple procedures and use of invasive devices distorting the anatomical integrity-protective barriers of patients (intubation, mechanical ventilation, vascular access, etc.). In addition, several drugs may be administered, which also predispose for infections, such as pneumonia, e.g., by reducing the cough and swallow reflexes (sedatives, muscle relaxants) or by distorting the normal nonpathogenic bacterial flora (e.g., stress ulcer prophylaxis) [1]. Consequently, the ICU population has one of the highest occurrence rates of (nosocomial) infections (20-30% of all ICU-admissions) [2,3], leading to an enormous impact on morbidity, hospital costs, and often, survival [4-6]. According to the EPIC II 1-day prospective point-prevalence study (Extended Prevalence of Infection in Intensive Care) in 1,265 participating ICUs (75 countries worldwide), 51% of the 12,796 patients were considered infected, although no subdivision was made for hospital-acquired infections [7].

Along with the problem of nosocomial infection goes the burden of "multidrug" antimicrobial resistance (MDR). The ongoing emergence of resistance in the community and hospital is considered a major threat for public health. Due to the specific risk profile of its residents, the ICU also is deemed the epicenter of resistance development. The ICU has even been described as a factory for creating, disseminating, and amplifying antimicrobial resistance [8]. Both infection and MDR result in a considerable clinical and economic burden. As such, the presence of MDR boosts the deleterious impact of nosocomial infection [9]. Compared with infections not caused by MDR microorganisms, the additional cost of multidrug resistance in hospitalized patients with infections has been estimated at $6,000 to $30,000 (per patient) [10]. This burden of resistance, however, is probably more due to the higher rate of inappropriate empiric antimicrobial treatment associated with infections caused by MDR pathogens than with the virulence of particular MDR strains [11]. Yet, several studies analyzed the attributable mortality of MDR in some of the most important nosocomial pathogens, such as *S. aureus*, *Enterococci*, *Pseudomonas *spp., *Acinetobacter spp*. etc. [9]. Even when mortality figures were adjusted for confounding factors, such as disease severity, exposure time, underlying diseases, etc., mortality increased (up to fivefold) when the causal organisms were MDR [9,12,13].

## Spread of multidrug resistance

The emergence of MDR often is dedicated to excessive use of broad-spectrum antimicrobial agents (more than 60% of all ICU patients receive antibiotics during their stay [14]), but the epidemiology of MDR is much more complex and multifactorial in nature. During the past few decades, it appeared that some antibiotics have a higher risk of promoting antimicrobial resistance, e.g., third-generation cephalosporins, vancomycin, imipenem, and intravenous fluoroquinolones [8,15]. Other antibiotics have been used for decades and still barely caused resistance (e.g., colistin). Bonten and Mascini recognized four main forces behind the emergence and further spread of MDR microorganisms [16]: 1) induction of resistant stains; 2) selection of resistant strains; 3) introduction of resistant strains; and 4) dissemination of resistant strains. These alterable/relative forces should be especially considered as incentives to tackle the spread of antimicrobial resistance, especially because all microorganisms have their own mechanism and flexibility to become resistant depending on their ideal environment to tackle antimicrobial efficacy [16].

### Induction of resistant stains

Resistance of susceptible bacteria can occur during antimicrobial treatment, e.g., by mutations [16]. Quinolone and cephalosporin resistance in *Enterobacter *spp. may arise through this mechanism, but it is very unlikely that it causes methicillin or vancomycin resistance in *S. aureus *or *enterococci *in a single patient [16].

### Selection of resistant strains

Antimicrobial therapy may select and favor overgrowth of preexisting resistant flora [16]. Therefore, it is important to maintain the nonpathogenic (anaerobic) flora, e.g., in the gastrointestinal tract, to prevent overgrowth of gram-negative MDR microorganisms [16].

### Introduction of resistant strains

The growing community reservoir of MDR microorganisms also results in a rise of MDR microorganisms in the ICU, especially for species, such as methicillin-resistant *S. aureus *and vancomycin-resistant enterococci [16]. Healthcare workers often are carriers but also can be vectors (cross-transmission) [9,16]. In addition, an increasing number of patients is already colonized with resistant bacteria on admission in the ICU [16]. When colonization pressure with resistant strains is above a certain level, the risk of cross-transmission becomes extremely high and very difficult to overcome (inoculum effect) [8]. Thus, in countries with a high endemic level of resistance (e.g., MRSA), there is a real risk of antibiotic "spiral" [8].

### Dissemination of resistant strains

As in all microorganisms, suboptimal infection control also facilitates the spread of MDR microorganisms [16].

## Important resistant pathogens

During the past decades, a shift in the MDR dilemma has been noted from gram-positive to gram-negative bacteria, especially due to the scarceness of new antimicrobial agents active against resistant gram-negative microorganisms [17]. Among gram-positive organisms, the most important resistant microorganisms in the ICU are currently methicillin-(oxacillin-)-resistant *Staphylococcus aureus*, and vancomycin-resistant enterococci [17,18]. In gram-negative bacteria, the resistance is mainly due to the rapid increase of extended-spectrum Beta-lactamases (ESBLs) in *Klebsiella pneumonia*, *Escherichia coli*, and *Proteus mirabilis*; high level third-generation cephalosporin Beta-lactamase resistance among *Enterobacter *spp. and *Citrobacter *spp., and MDR in *Pseudomonas aeruginosa, Acinetobacter spp., and Stenotrophomonas maltophilia *[18]. Together with the rise of difficult-to-treat MDR infections, several other types of infections become more difficult to treat, whereof the anaerobic *Clostridium difficile *spp., and fungal infections will be described briefly.

### Methicillin-resistant Staphylococcus aureus (MRSA)

Although methicillin-resistant *Staphylococcus aureus *(MRSA) is already dethroned as the most feared MDR microorganism by the emerging MDR gram-negatives, it remains difficult to eradicate. Being endemic in numerous hospitals worldwide, MRSA is still among the most important causes of hospital-associated bacterial infections [15]. The driving force of resistance in MRSA is cross-contamination and admission of already colonised patients to the ICU [12,16]. Due to resistance to several other (beta-lactam) antimicrobials, treatment of MRSA often relies on vancomycin [18]. Yet, transmission of resistance plasmids from enterococci to *staphylococci *resulted in an increase of high-level, vancomycin-resistance among *S. aureus *[15,18]. The National Nosocomial Infection Surveillance (NNIS) study reported a rise of MDR in *S. aureus *from 3% in the early 1980s to 53% at the beginning of the 21^st ^century [9,19]. According to the EPIC II study the percentage of methicillin-resistant isolates of *S. aureus *was approximately 50% in Western and Eastern Europe, but approximately 65% in the Americas [7]. The epidemiology of MRSA is extremely sensitive to changes in admission prevalence and failures in infection control, which are only partially influenced by reductions in antibiotic use [16]. Previous stay in hospital or long-term care facilities, ICU stay, intravascular devices, prior or prolonged antibiotic therapy, chronic underlying conditions, surgical wounds, advanced age, and cross-contamination are the most important risk factors for MRSA colonization and infection [15].

### Vancomycin-resistant enterococci (VRE)

Enterococci have a remarkable ability to adapt to environmental changes and acquire antimicrobial resistance, leading to multiple vancomycin-resistant phenotypes, but also resistance to ampicillin, aminoglycosides, and other beta-lactam antibiotics [18]. As in MRSA, cross-contamination and admission of already colonized patients to the ICU are important causes for the spread of VRE [12,16]. In addition, VRE genes can be transmitted to other species through plasmids [18]. Enterococci also are among the most frequent causes of hospital-acquired infections and the rise of VRE during the past decades has been remarkable (from < 1-25% since the early 1980s) [9,15,20]. According to the EPIC II study, rates of vancomycin resistance among enterococcal isolates were approximately 33-40% in Western Europe, Eastern Europe, and Asia but approximately 50% in the Americas and Oceania [7]. The rate of vancomycin resistance varies among enterococcal spp., and is highest in *E. faecium*, of which >80-85% also is resistant to ampicillin and penicillin and >50% to high-level gentamicin [21]. VRE favors the highly compromised patient, with as most important independent risk factors for VRE colonization or infection, previous antibiotic exposure (vancomycin, cephalosporins, or agents with anaerobic activity), enteral feeding, cross-contamination, and prolonged hospital stay [15,22].

#### Enterobacteriaceae

In contrast to MRSA and VRE, for which a single antibiotic indicates the resistance phenotype of interest, MDR is usually more difficult to define in gram-negative bacilli, due to cross-transmission of resistance characteristics and the wide range of antimicrobials not active against (most) gram-negative microorganisms [18,22]. Resistance of gram-negative bacteria is consequently emerging in the hospital setting, especially through the production of extended spectrum beta-lactamases (ESBLs), in particular by *E. coli *and *Klebsiella *spp. and *Proteus *spp. [13,18,23,24]. ESBLs are plasmid-mediated, and their potential for transfer makes effective control and treatment difficult, which has resulted in endemic and epidemic outbreaks [23]. ESBLs were first recognized in the early 1980s among *K. pneumoniae *in Europe, and its prevalence has increased dramatically in the ICUs and in the community (e.g., extended care facilities), with now more than 500 different types of beta-lactamases identified from clinical isolates [21,25]. A decreasing efficacy in gram-negatives of third-generation cephalosporins, carbapenems, and fluoroquinolones has been described [18,21]. The development of quinolone resistance needs only two mutations, e.g., expression of inducible cephalosporin-resistance in *Enterobacter *spp. [16]. Especially the production of *Klebsiella pneumonia *carbapenemase (KPC) by *Enterobacteriaceae *becomes problematic, because KPC beta-lactamases result in decreased susceptibility or resistance to virtually all beta-lactam antibiotics; and many strains of *Enterobacteriaceae *were already resistant to a wide range of non-beta-lactam antibiotics [26].

Since 2000, the already ubiquitous *E. coli *has emerged as major ESBL producing organism. In contrast to *Klebsiella *spp., which is usually involved in nosocomial/ICU infections, ESBL-producing *E. coli *appeared more frequently in community-acquired infections, especially in certain areas in Asia [25]. In 2007, already 79% of *E. coli *isolates collected in India, were positive for ESBLs, with an almost identical prevalence in both hospital and community [25,27].

Reported risk factors for infection/colonisation with ESBL are prior use of antimicrobials, ICU stay, indwelling devices, increased illness-severity, prolonged (ICU-) hospitalization, emergency intra-abdominal surgery, mechanical ventilation, and residence in nursing homes [21,25].

#### Pseudomonas/Acinetobacter

Nosocomial isolated strains of *Pseudomonas *and *Acinetobacter spp.*, are frequently resistant to a broad range antibiotics. Their prevalence appears to be increasing worldwide, especially as cause of ventilator-associated pneumonia or in high-risk populations, such as patients with severe burn injury [22,28]. The respiratory tract is the most important source of *Pseudomonas *isolates, followed by wounds, urine, and the bloodstream [21]. *Pseudomonas *is intrinsically resistant to most antibiotics; most active agents are carbapenems, piperacillin, cefepime, ceftazidime, ciprofloxacin, amikacin, and tobramycin [21]. Antimicrobial resistance develops rapidly under selection pressure, and multiple mechanisms are responsible: (hyper-)production of enzymes, such as beta-lactamases and DNA-gyrases, active efflux pumps, and permeability changes [21]. The NNISS study showed 27% fluoroquinolone-resistance in *Pseudomonas *isolates in the ICU and 18% to imipenem [19]. Furthermore, cross-resistance between fluoroquinolones and other antibiotic agents, such as piperacillin-tazobactam, ceftazidime, and tobramycin is a frequent problem [21,29].

*Acinetobacter *spp. are inheritably resistant to cephalosporins, penicillin's, and aminoglycosides, and especially cause opportunistic infections in critically ill patients [21]. Some strains *of A. baumannii *have been detected that are resistant to all antibiotics [21,30]. They rarely cause community-acquired infections and are more common in tropical countries [21].

#### Clostridium difficile

*Clostridium difficile *spp. are among the most difficult microorganisms to eradicate in the environment [31]. Its' spores cannot be destroyed by antiseptics, such as alcohol, so hand washing with water and soap is essential. Disease severity ranges from mild diarrhea to fulminant pseudo-membranous colitis; *Clostridium *spp. are actually responsible for almost all antibiotic-associated pseudo-membranous colitis [32]. The incidence is rather low although rising, and several outbreaks have been described [31]. Due to a reduced susceptibility to metronidazole, antibiotic treatment also becomes more difficult [33]. Most important risk factors for *Clostridium*-associated diarrhoea antimicrobial therapy, older age (>65 years), antineoplastic chemotherapy, and length of hospital stay [32]. Other interventions with high-risk associations are enemas, nasogastric tubes, gastrointestinal surgery, and antiperistaltic drugs [32].

#### Candida infections

Due to their immunocompromised status, patients in the ICU are at risk of invasive candidiasis [34,35]. The problem of MDR in candidiasis merely results from a shift in etiology from mainly *C. albicans *to non-*albicans *spp., such as the intrinsically fluconazole-resistant *C*. *krusei *or the dose-dependent susceptible *C. glabrata *[36]. Leroy et al. found that almost half of the invasive candida infections in the ICU (N = 300) were due to non-albicans species and reduced susceptibility to fluconazole was observed in 17% of all *Candida *isolates [37]. A prospective study on candidemia of the same authors (N = 135) failed to find pertinent risk factors predicting non-*albicans *spp. [38], although two other studies found an association between non-*albicans *candidemia and azole treatment [36], female gender [39], and also with (duration of) the use of central venous catheters [36,39].

### Resistance in the ICU and abroad

Although the ICU can be considered as the epicenter of MDR, the past decades a notable shift has taken place to general wards, nursing homes, and even the broader community. The European Antimicrobial Resistance Surveillance System (EARSS), which collected data on antimicrobial resistance in spinal fluid and blood samples in 31 countries, showed a wide variation in MDR among and within different countries [40]. Especially MRSA continues to spread, although a stabilization (or even decrease) is noted in high endemic countries. However, such data are generally limited to hospital settings, whereas the total burden of MRSA in the broader community remains a blind spot. Besides MRSA, VRE, and the MDR *Enterobacteriaceae *(in particular *E. faecium *and *E. coli*) remain important challenges for infection control [40].

Infection control measures have important implications for daily practice, because more and more patients are already colonised or infected with MDR microorganisms on arrival in the ICU [41]. Therefore, broad empiric coverage may be needed in several patients; in particular those patients with prolonged hospitalizations (not only ICU). Until several years ago, this strategy was only needed for patients with classic risk factors for MDR, namely >1 week in ICU, and previous exposure to antimicrobial agents. A recent Belgian multicenter study showed that these classic risk factors lost their predictive value as in 40% of infected patients without risk factors; the causative pathogen was MDR [41].

## Solutions?

Because the pipeline of new antibiotics is running dry [17], major efforts are needed to slow down the rising problem of MDR. The Centres for Disease Control recommends four strategies for health care settings: 1) prevent infections; 2) diagnose and treat infections; 3) prudent and rational use of antimicrobials; and 4) prevent transmission [8,9]. Joined efforts of healthcare providers, hospital administrators, policy makers, and patients will certainly be necessary (up to an international level) to reduce and optimize the overall antibiotic consumption. This should especially affect those most vulnerable patients, at highest risk for fatal outcomes, namely those in the ICU, because local efforts limited to the ICU will have too little impact. "Antibiotic stewardship," or the optimization of antibiotic usage for therapy and prophylaxis, is certainly a keystone to tackle this problem [42,43].

Yet, there is a wide variation in current practice, partially due to a lack in evidence on the best strategies to cope with prevention of infection and spread of MDR. Although several measures in infection control are widely accepted, hand hygiene for example, there exist some controversy about others, mostly more complex interventions. Frequently infection prevention measures are subject to a debate that is concentrated on the triangle: economic issues vs. health vs. evidence [44]. Restriction of antibiotic consumption by a sensible hospital drug policy and promotion of a more rational use of antibiotics should halt the rising of MDR [42]. Therefore, additional efforts are needed to improve education and training, for example, by implementing guidelines in infection control and antibiotic prescription. Yet, surveillance and monitoring of trends in MDR, with timely updates of local susceptibility data, should be implemented as well [42,43].

In the battle against MDR, several behavioral changes have been proposed to reduce or to improve antimicrobial therapy in community, long-term care facilities, and hospitals. In the ICU-specific strategies, such as antimicrobial cycling and de-escalation schemes have been proposed, although there often is a strident contrast between evidence and expert opinion [45,46]. Even the high ("standard") use of antimicrobials in the ICU has been questioned lately [44]. Especially in ICU patients with severe sepsis, use of wide-spectrum antibiotics is believed to be necessary, because these patients are supposed to have little margin for error in choice of therapy. Therefore, the initial selection of antibiotics should cover all likely pathogens [44]. Peculiarly, even this hypothesis is based on only a few studies, usually retrospective. This stands in contrast to high levels of "inappropriate" early antibiotic therapy, which does not always lead to tremendously increased mortality rates [47].

When it concerns prescription of antibiotic therapy, the clinician must balance between optimizing the odds of survival for the individual patient and minimizing the microbial selection pressure. A study of Lipsitch et al. [46] concluded that "*use of an antibiotic for which resistance is not present will be positively associated at the individual level with carriage of bacteria resistant to another antibiotic but negatively associated at the population level with the prevalence of resistance to the other antibiotic"*. Routine surveillance cultures to steer empiric antibiotic therapy may result in higher rates of initial appropriate therapy while including a substantial potential in the savings of last-line antibiotic agents [8,48]. Another practice that clearly showed to be beneficial in several studies is selective digestive decontamination, also in reducing the overall number of infections [49-52]. This technique mainly eradicates potentially pathogenic gram-negative aerobic bacteria in the gastrointestinal tract through a combination of enteral and parenteral antimicrobial therapy. This approach is steered by additional efforts in infection control, including the use of routine surveillance cultures. Several studies showed clear benefit [51], but despite this, only a few hospitals adopted this strategy in daily practice. A drawback of selective digestive decontamination is that it may promote overgrowth of MDR gram-positive bacteria, such as MRSA. As such, it is preferably used in setting with a very low baseline prevalence of MRSA [53].

Thus, species-specific impact of preventive measures certainly depends on the local epidemiology and resistance levels. Therefore, prevention programs should be tailored to the local epidemiology and organized hospital-wide and not only localized to the ICU [8]. A team approach should be preferred, including ICU physicians and nurses, but also the infection control team, and the team especially needs a strong cooperation with infectious disease specialists and clinical microbiology teams [54-56]. Several care bundles, or "packages of interventions," have been promoted instead of single efforts to prevent infections, although these care bundles are rarely based on solid evidence [44].

## Conclusions

Infections due to MDR microorganisms are a rising problem, especially in the ICU where even sensitive pathogens already cause additional morbidity, mortality, and hospital costs. Therefore, additional efforts are needed in the future to win this battle. Constant evaluation of current practice on basis of trends in MDR and antibiotic consumption patterns is essential to make progress in this problematic matter.

## Competing interests

The authors declare that they have no competing interests.

## Authors' contributions

NB, DV, and SB contributed to draft the manuscript and approved the final version.
